# An Optimized Facile Procedure to Synthesize and Purify Allicin

**DOI:** 10.3390/molecules22050770

**Published:** 2017-05-10

**Authors:** Frank Albrecht, Roman Leontiev, Claus Jacob, Alan J. Slusarenko

**Affiliations:** 1 Department of Plant Physiology, RWTH Aachen University, D-52056 Aachen, Germany; frank.albrecht@rwth-aachen.de (F.A.); roman_leontiev@gmx.de (R.L.); 2 Division of Bioorganic Chemistry, School of Pharmacy, Campus B2 1, University of Saarland, D-66123 Saarbruecken, Saarland, Germany; c.jacob@mx.uni-saarland.de

**Keywords:** allicin, *Allium sativum*, diallyl-disulfide, catalytic oxidation, reactive sulfur species, dipropyl-disulfide, thiosulfinate

## Abstract

Allicin is a reactive sulfur species (RSS) and defence substance from garlic (*Allium sativum* L.). The compound is a broad-spectrum antibiotic that is also effective against multiple drug resistant (MDR) strains. A detailed protocol for allicin synthesis based on diallyl-disulfide (DADS) oxidation by H_2_O_2_ using acetic acid as a catalyst was published in 2001 by Lawson and Wang. Here we report on improvements to this basic method, clarify the mechanism of the reaction and show that it is zero-order with respect to DADS and first-order with respect to the concentration of H_2_O_2_. The progress of allicin synthesis and the reaction mechanism were analyzsd by high-performance liquid chromatography (HPLC) and the identity and purity of the products was verified with LC-MS and ^1^H-NMR. We were able to obtain allicin of high purity (>98%) and >91% yield, with standard equipment available in any reasonable biological laboratory. This protocol will enable researchers to prepare and work with easily and cheaply prepared allicin of high quality.

## 1. Introduction

The sulfur-containing compound allicin (2-Propene-1-sulfinothioic acid *S*-2-propenyl ester, or diallyl-thiosulfinate, DATS) is produced in damaged tissue of garlic (*Allium sativum*), ramsons (*Allium ursinum*), and hooker chives (*Allium hookeri*) and gives these plants their typical odours [1]. Garlic is highly valued in the cuisines of many nations because of its excellent flavour and its pungent smell. Additionally, it has long been believed that allicin, or at least garlic consumption, is beneficial to health [2]. In 1944 Cavallito and Bailey demonstrated that allicin inhibited the growth of *Staphylococcus aureus* and other bacteria in liquid culture [3]. Furthermore, allicin was shown to induce apoptosis, often selectively, in mammalian cancer cells cultured in vitro [4,5], in intact tissues in vivo [6], and in cells of yeast (*Saccharomyces cerevisiae*), a model fungal eucaryote [7]. These properties turn allicin into a highly interesting compound for clinical investigations. Stoll and Seebeck first reported the synthesis of allicin in 1947, but without specifying experimental details [8]. Their chemical synthesis of allicin was based on the oxidation of diallyl-disulfide (DADS) by peracetic acid as a mild oxidizing agent. A more detailed protocol of this basic method was published by Lawson and Koch in 1994 and Lawson and Wang in 2001 [9,10]. Other methods to synthesize allicin utilizing magnesium monoperoxyphthalate [11] or chloroperbenzoic acid have also been reported [12,13]. Nevertheless, it is still challenging to obtain pure allicin in acceptable yields.

In the original protocol, DADS was stirred into a mixture of acetic acid and H_2_O_2_ and incubated at room temperature (RT) for 4 h with constant stirring. The reaction was stopped by adding five volumes of water and extracted with dichloromethane (DCM) to retrieve allicin along with unreacted DADS, some acetic acid, and DCM-soluble reaction byproducts. The lipophilic undissociated acid catalyst in the DCM phase was neutralized with aqueous sodium carbonate solution which facilitated partitioning of the hydrophilic sodium acetate generated into the aqueous phase. DCM was removed by rotary evaporation at RT at reduced pressure to yield an oily residue of allicin, unreacted DADS, and byproducts. Further purification of allicin was based on the differential partitioning of the constituents of the oily residue between *n*-hexane and an aqueous phase (two washes). Unreacted DADS and some allicin accumulated in the *n*-hexane phase, but allicin, which is more polar than DADS, concentrated to some extent in the aqueous phase. The separation method was inefficient, however, and allicin losses occurred at this stage. Finally, the allicin-containing aqueous phase was partitioned against DCM to isolate allicin and dried over anhydrous CaSO_4_. Allicin was obtained as an oily residue after evaporation of the DCM under reduced pressure at RT.

This synthesis consists of at least two reaction steps. Firstly, the organic peracid is formed by oxidation of the organic acid by H_2_O_2_. Secondly, DADS is oxidized by the peracid, thus regenerating the parent organic acid. It has been reported that peracids, such as performic and peracetic acids, are adequately soluble in the organic phase [14], but DADS is immiscible with the aqueous H_2_O_2_ solution and the reactions therefore take place in a two phase system.

In the optimized method described in this paper we used a formic acid catalyst instead of acetic acid, which enabled us to carry out the reaction at 0 °C under more controlled conditions and we systematically varied the concentrations of the reactants, while following the progress of the reaction using HPLC. Furthermore, we developed a silica gel column chromatography protocol for allicin purification which avoided the losses associated with the original solvent partitioning procedure.

**Scheme 1:**
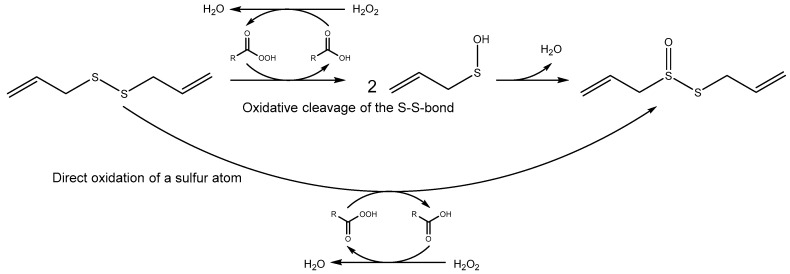
Allicin synthesis from diallyl-disulfide (DADS) through oxidation by a peracid generated with H_2_O_2_ in the reaction mixture. The organic acid serves as an intermediate catalyst.

A reaction mechanism for Stoll and Seebeck’s synthesis was postulated by Nikolic et al. [15] proposing oxidative cleavage of the S–S bond in DADS by hydroxyl-radicals generated from the acidic H_2_O_2_ to give allyl-sulfenic acid which condenses to yield allicin (Scheme 1). In contrast, an alternate mechanism, namely direct oxidation of one of the S-atoms in DADS without oxidative cleavage of the S–S bond, is also plausible (Scheme 1). Here we provide data supporting an oxidative cleavage mechanism and condensation of two sulfenic acid molecules to yield allicin, but without a need for hydroxyl-radicals.

## 2. Results and Discussion

### 2.1. Comparison of DADS Oxidation Catalyzed by Acetic Acid or Formic Acid

**Figure 1:**
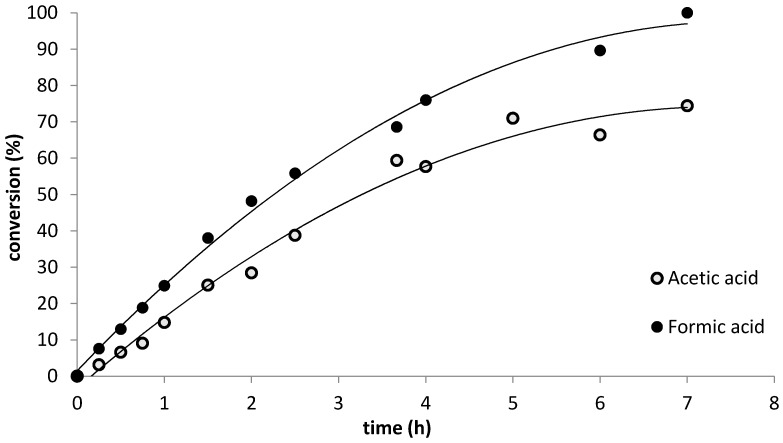
Kinetics of allicin synthesis. The oxidation of DADS to allicin was catalyzed by either acetic acid at 20 °C or formic acid at 0 °C. All reactions took place in sealed 2 mL reaction tubes in temperature-controlled rotary shakers and via continual shaking at 1200 rpm to ensure optimal mixing. The products were separated by HPLC and quantified with a UV detector at 254 nm. Reaction progress was followed by calculating the percentage ratio of actual allicin yield divided by the theoretical maximum yield to indicate the percentage of conversion during the course of the reaction.

Preliminary experiments substituting formic acid for acetic acid at RT resulted in rapid overheating of the reaction mixture accompanied by massive byproduct formation, therefore, we carried out the formic acid catalyzed allicin syntheses at 0 °C. The progress of the oxidation reactions was followed by HPLC analysis; i.e., disappearance of the DADS peak and appearance of the allicin peak. In a first attempt, we withdrew small samples of the proceeding reaction, diluted them with methanol and measured the amount of allicin and DADS. This approach was not reliable, however, due to the fact that the reaction mixture was an emulsion. It was, therefore, difficult, despite thorough mixing, to guarantee the same distribution of content in withdrawn samples and the remainder of the reaction mix in the flask. Measurements confirmed these concerns and showed unrealistic kinetics (data not shown). Therefore, in a second approach, the reaction was carried out in several parallel aliquots on a micro-scale and each aliquot was diluted with methanol as a whole to give a single data point. Thus, every time point presented in Fig. 1 shows an independent parallel reaction run. Reaction progress was followed by calculating the percentage ratio of actual allicin yield divided by the theoretical maximum yield (100% of DADS converted to allicin) to indicate the percent conversion during the course of the reaction. Despite the lower reaction temperature, allicin was formed more rapidly and to a greater yield (78% conversion by 4 h) with formic acid as catalyst than with acetic acid (58% conversion at 4 h) (Figure 1).

**Figure 2:**
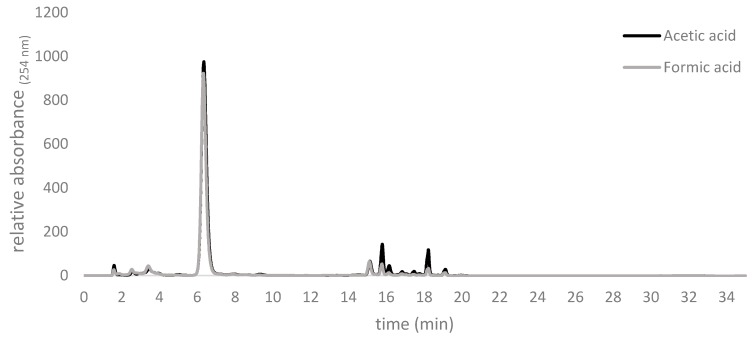
Comparison of representative end point traces of the products of routine allicin syntheses with either acetic or formic acids.

Byproducts detectable by HPLC and presumably arising via decomposition, were observed increasingly with incubation times longer than 4 h. Quantitatively slightly lower amounts of byproducts were observed at 0 °C with formic acid as a catalyst than with acetic acid at 20 °C (Figure 2).

Lesser byproduct formation using formic acid at 0 °C as shown in Figure 2 may be explained by allicin’s increased reactivity and inherent instability at higher temperatures. The instability of allicin at higher temperatures was reported to be increased by hydrophobic solvents such as any residual DADS [16]. For those reasons, the reaction should be stopped at the latest after 4 h, by adding five volumes of H_2_O, even though conversion is incomplete. Furthermore, if not tempered to 20 °C different RTs will lead to different kinetics for the reaction and the need for new calibrations. Therefore, we propose that it is advantageous for reasons of increased yield and reaction consistency to use formic acid as a catalyst and to carry out the reaction on ice at 0 °C.

### 2.2. Reaction Order with Respect to Individual Reactants

**Figure 3:**
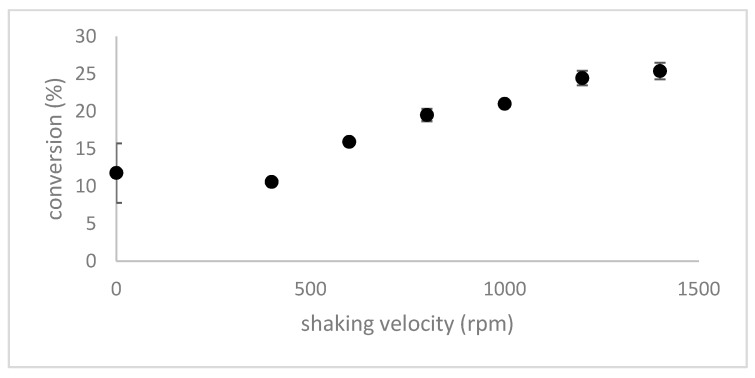
The effect of shaking velocity on the reaction rate. Micro-scale reactions using a pre-incubated mixture of H_2_O_2_ and formic acid (the reagent mix was stored two weeks at 4 °C) and shaken at various speeds at 0 °C to mix the reactants. Reactions were stopped after 5 min. The maximum reaction rate was achieved by 1200 rpm and statistical analysis according to the Holm-Sidak method showed no significant difference between the rates at 1200 rpm and 1400 rpm.

The kinetics shown in Figure 1 not only reveal a faster reaction when formic acid is used as a catalyst, but also give information about the reaction order. Thus, after 2 h—48%, after 4 h—76% and after 6 h—90% of the DADS was converted to allicin. This is an approximate halving of the amount of DADS every 2 h indicating that the overall reaction followed first order kinetics. We investigated the reaction kinetics in more detail and showed that the shaking conditions for the two phase reaction were a limiting factor for the reaction speed (Figure 3). The reaction rate can be seen to increase proportionally up to 1200 rpm, which was thus chosen as the routine shaking velocity for micro-scale synthesis reactions.

Since the oxidation of DADS occurs in the organic DADS phase by peracid dissolved in it, varying the amount of DADS in the reaction mix does not actually affect its concentration relative to the peracid. Therefore, the reaction follows a pseudo-zero-order kinetic with respect to DADS. Furthermore, it was observed that with pre-incubation of H_2_O_2_ and formic acid a higher rate of DADS conversion to allicin was achieved than when all reactants were mixed at once, suggesting that peracid formation was a rate-limiting step. This aspect will be investigated in the next section.

**Figure 4:**
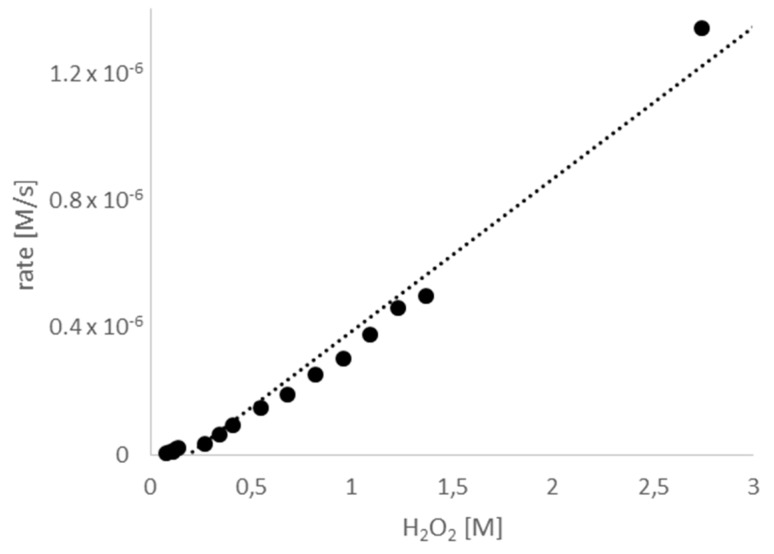
The rate of allicin formation in relation to the H_2_O_2_ concentration. Micro-scale reactions were performed with varying H_2_O_2_ concentrations. The reactions were stopped after 30 min, when turnover of H_2_O_2_ was between 14% (start conc. 2.74 M) and 0.9% (start conc. 0.082 M), the linear progression indicates that neither H_2_O_2_ nor DADS were limiting in the reaction.

In contrast, varying the concentration of H_2_O_2_ affected the rate of product formation. As shown in Figure 4 there was a linear relationship between the concentration of H_2_O_2_ and product formation. Therefore, the reaction follows first-order kinetics with respect to the concentration of H_2_O_2_.

### 2.3. Preformation of Performic Acid

**Figure 5:**
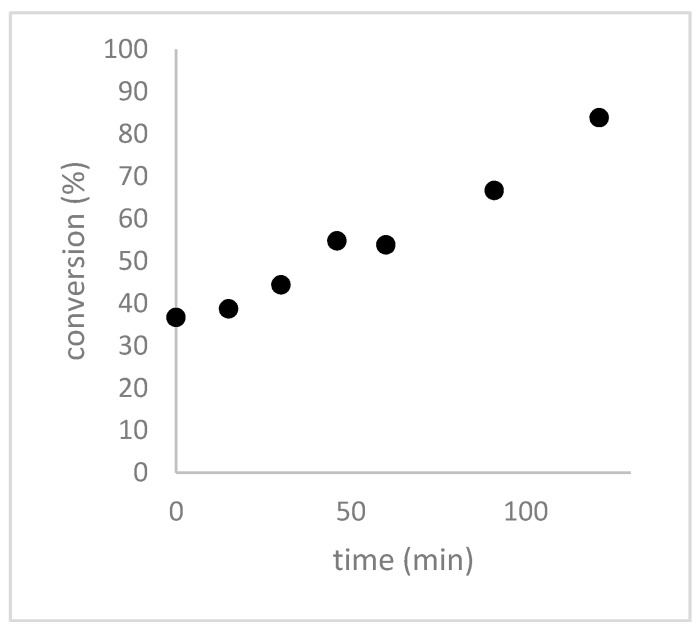
Effect of allowing 3 h at 0 °C for the preformation of performic acid on the rate of conversion of DADS to allicin. H_2_O_2_ and formic acid were mixed according to the micro-scale reaction procedure and incubated on ice for 3 h before DADS was added. The reactions were stopped with methanol, separated by HPLC and quantified with a UV detector at 254 nm.

We observed that when H_2_O_2_ and formic acid were mixed 3 h before the addition of DADS, a 37% conversion of DADS to allicin was observed within seconds and that the conversion was >80% complete after 120 min (Figure 5).

**Figure 6:**
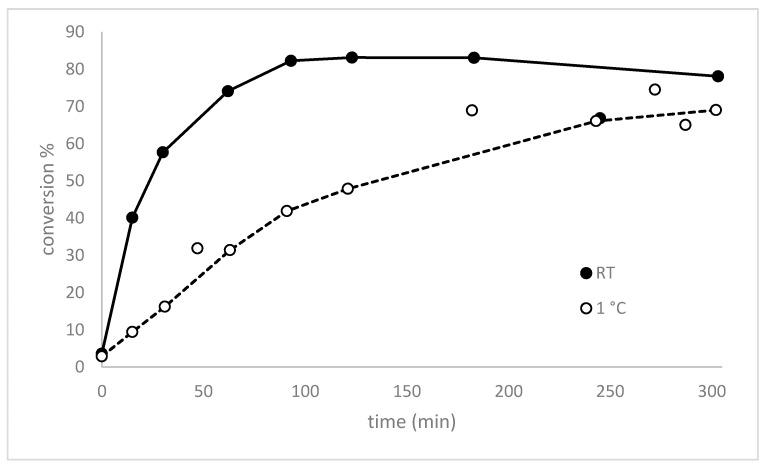
The effect on the pre-incubation time for performic acid formation on the rate of DADS conversion to allicin. H_2_O_2_ and formic acid were mixed according to the micro-scale reaction procedure and incubated at room temperature or 1 °C for the indicated times before DADS was added. The reactions were stopped after 5 min by addition of methanol, separated by HPLC and quantified with a UV detector at 254 nm. A maximal turnover was reached after 100 min at RT, indicating that maximum performic acid formation was achieved after that time.

Without preformation of the performic acid the reaction needs ~1.5 h to reach >35% conversion and showed >80% conversion only after ~4 h (Figure 1). This illustrates clearly that the formation of performic acid is rate limiting for allicin synthesis, therefore, we decided to investigate systematically the effect of the pre-incubation time of H_2_O_2_ and formic acid on the conversion rate of DADS to allicin in order to optimize this step in the protocol (Figure 6).

We investigated the turnover of DADS to allicin depending on the pre-incubation time of the standard amounts of H_2_O_2_ and formic acid at 0 °C and at RT. The maximum turnover was reached between 100 and 180 min, followed by decrease of the turnover. Our observations are in accordance with those of Filippis et al. [17] who showed, that the formation of performic acid was a slow temperature dependent mechanism. In their experimental setup the maximum turnover of 25% of the H_2_O_2_ was reached after 100 min at 30 °C; thereafter the concentration started to decrease due to performic acid decomposition. Thus, in our optimized allicin synthesis protocol we recommend a 100 min pre-incubation step at RT to pre-form the performic acid.

### 2.4. Influence of Formic Acid Concentration and Amount of H_2_O_2_ on the Conversion of DADS to Allicin

**Figure 7:**
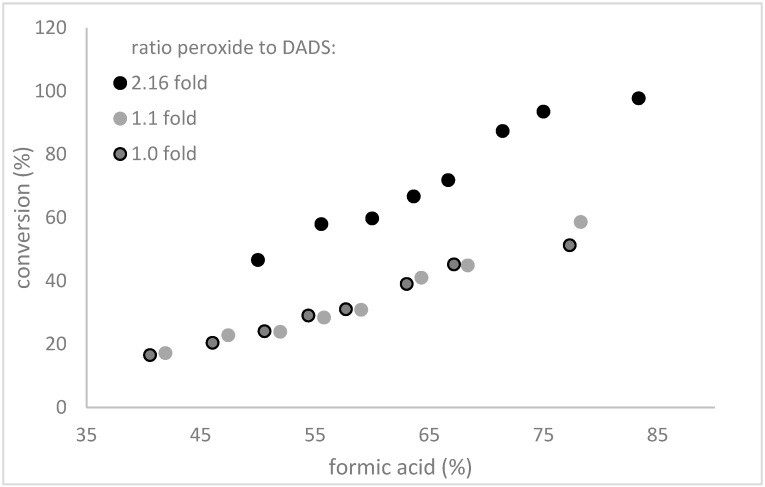
The influence of formic acid concentration and amount of H_2_O_2_ on the conversion of DADS to allicin. In a series of microscale reactions, the amount of H_2_O_2_ was varied three times from 1 mol H_2_O_2_ per mol DADS to 2.16 mol H_2_O_2_ per mol DADS, indicated with different data point styles. The concentration of formic acid was also varied between 40% and 85% in the reaction. All H_2_O_2_-formic acid mixtures were pre-incubated for 3 h on ice. The lower black data points (50% formic acid; 47% conversion) are conform to standard micro-scale reaction conditions. The figure shows that higher concentrations of acid and peroxide enhance the speed of the reaction.

Having established that preformation of performic acid greatly enhanced the conversion of DADS to allicin, we analysed the procedure with respect to formic acid and H_2_O_2_ concentrations. In micro-scale reactions higher amounts of acid and H_2_O_2_ increased the rate of the reaction (Figure 7), but when the reaction volume was scaled up this effect was less pronounced. See Section 2.5.

### 2.5. Accelerated Allicin Synthesis

**Figure 8:**
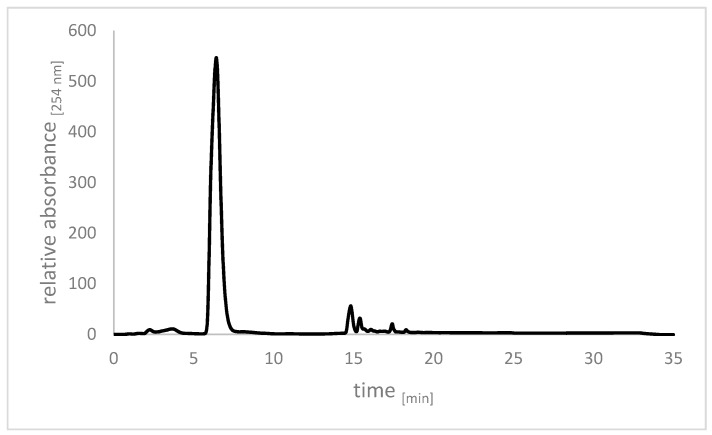
Chromatogram of crude allicin after synthesis using pre-formed performic acid. The reaction was extracted with dichloromethane (DCM) and the solvent was removed by rotary evaporation. The crude product already showed good purity.

Not all of the advantages observed by altering parameters in the micro-scale reactions were completely transferable to scaled up reactions. The reaction speed was not as high as on the microscale and the formation of byproducts became more prevalent (data not shown). The latter are problems which could be due lesser mixing inefficiency and emulsion formation on the larger scale. In order to avoid inadequate mixing, we used methanol to combine the two phases and prevent emulsion formation (Section 3.2.3). In this way, a conversion of >98.46 ± 0.45% in just 15 min was achieved (Figure 8).

### 2.6. Purification of Allicin

After quenching the reaction by addition of H_2_O, the reaction mixture consists of allicin, DADS, formic acid, H_2_O_2_, and byproducts. The organic compounds were extracted by partitioning against either dichloromethane (DCM) or diethyl-ether. In the Lawson method remaining acetic acid was removed by washing the organic phase with Na_2_CO_3_ solution or extracting several times with water. This, however, leads to a loss of allicin, some of which partitions into the aqueous phase. A further advantage of using formic acid as a catalyst becomes apparent here. Formic acid is more volatile than acetic acid and, therefore, more easily removable under reduced pressure at room temperature, thus switching to evaporation instead of washing and, hence, avoiding the Na_2_CO_3_ washing step.

**Figure 9:**
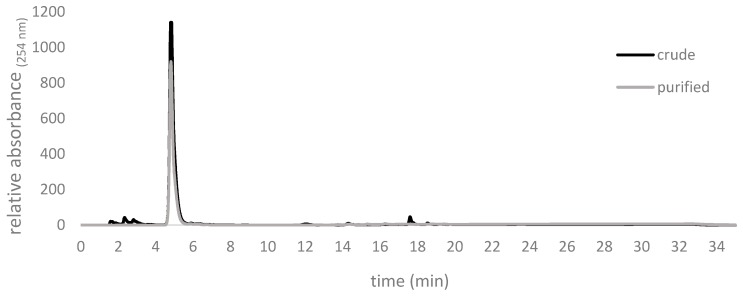
Chromatograms of crude allicin after synthesis using formic acid as a catalyst and rotary evaporation and afterwards purification by silica gel chromatography. The crude product contained impurities with retention times of about 2 min and 18 min, respectively, whilst the purified product showed >98% purity. Respresentative traces of our repeated routine syntheses are shown.

After rotary evaporation, separating allicin, DADS and byproducts is challenging, due to the similar physical properties of these compounds. The Lawson method partitioned repeatedly between *n*-hexane and water to accumulate allicin in the aqueous phase. The calculated log*P* values (clog*P*) of allicin (1.35), DADS (2.95), and probable byproducts such as vinyl-dithiine (2.69) and ajoene (1.97) (Chemdraw, see Section 3) indicate that allicin is the least hydrophobic molecule. Nonetheless, repeated extractions lead to further losses of allicin. To circumvent this we used silica gel chromatography to separate allicin from the other compounds (Figure 9). The structure of the final product was confirmed and the purity was determined by ^13^C-NMR and ^1^H-NMR, respectively.

### 2.7. Reaction Mechanism

**Scheme 2:**
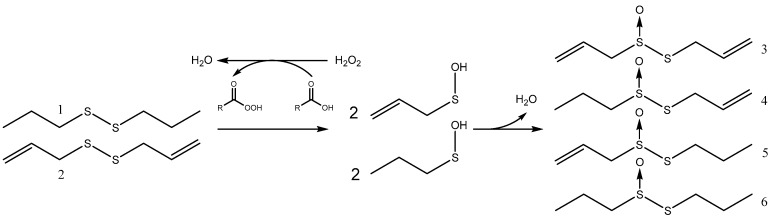
The basic reaction for mixed thiosulfinate synthesis from DADS and DPDS by oxidation by peracid generated with H_2_O_2_ in the reaction mixture.

We reasoned that if allicin synthesis proceeded by direct oxidation of DADS (1) without oxidative cleavage of the S–S bond, then a mixture of DADS and dipropyl-disulfide (DPDS, 2) would yield allicin (DATS, 3) and dipropyl-thiosufinate (propicin, DPTS, 6) only. In contrast, if oxidative cleavage of the S–S bond occurred, then mixed allyl-propyl thiosulfinates should be further products because of random condensation of the respective sulfenic acids (Scheme 2). Thus, formation of *S*-allyl-propane-1-sulfinothioate (4) and *S*-propyl-prop-2-ene-1-sulfinothioate (5) would be predicted.

**Figure 10:**
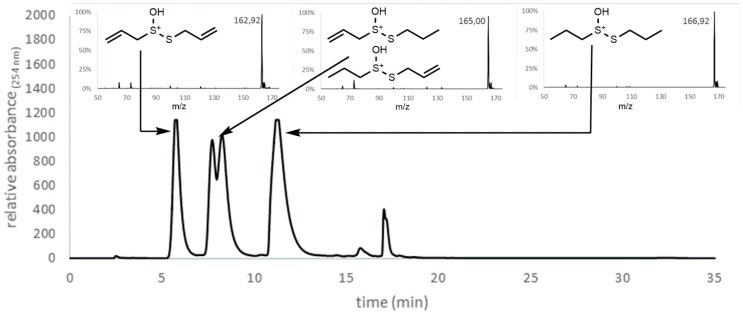
Synthesis of mixed thiosulfinates. According to Scheme 2 a reaction mechanism via the formation of sulfenic acid would be expected to produce mixed thiosulfinates from a mixture of reacting alkyl disulfides. The chromatogram was obtained by HPLC and quantified with a UV detector at 254 nm. Masses were identified in a separate LC-MS analysis using the same column and gradient. Insets show the *m*/*z* ratios in MS-traces and the respective structures of the major ions. The masses obtained fit to the expected molecules: 6 min, 162.92 Da—allicin (3); 8 min, 165.00 Da—*S*-allyl-propane-1-sulfinothioate (4) and *S*-propyl-prop-2-ene-1-sulfinothioate (5); 11 min, 166.92 Da—propicin (6).

**Scheme 3:**
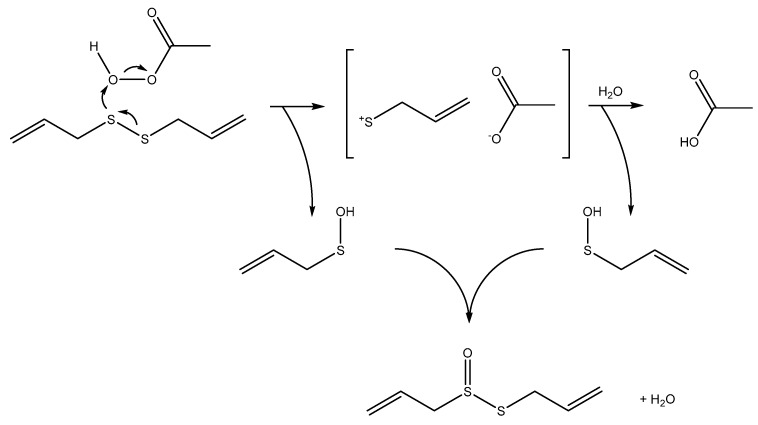
The oxidation of disulfides proceeds via the formation of an allyl cation and an allylsulfenic acid in the first step. In a second step the allyl cation reacts with water to form a second allylsulfenic acid. The resulting sulfenic acids condense in a third step to form allicin.

A mixture of DADS (1) and DPDS (2) was oxidized by performic acid, as described in Section 3.2.4. After the reaction was quenched products were extracted with DCM. LC-MS analysis of the crude extracts showed, in addition to single peaks at 6 min and 11 min, which were identified as allicin (3) and propicin (6), respectively, a double peak at 8 min from the mixed thiosulfinates (4,5). Data in Figure 10 are a combination of the UV absorption chromatogram detected by HPLC and the mass signals detected with LC-MS. These data indicate that oxidation of alkyl disulfides to thiosulfinates by peracids proceeds via oxidative cleavage of the S–S bond, but does not formally rule out parallel direct *S*-atom oxidation without S–S bond cleavage. Therefore, we suggest the reaction mechanism shown in Scheme 3.

## 3. Materials and Methods

### 3.1. Materials

DADS (≥80%) was purchased from Sigma Aldrich (Munich, Germany). DPDS (98%) was purchased from Sigma Aldrich. Formic acid (≥98%, p.a.) was purchased from Carl Roth (Karlsruhe, Germany). H_2_O_2_ (30%) was purchased from Merck (Darmstadt, Germany). Acetic acid (100%, p.a.) was purchased from Carl Roth. TLC was performed using Merck TLC Silica gel 60 F_254_ with concentration zone. Solvent A (*n*-hexane ≥99% p.s.) was purchased from Carl Roth. Solvent B (ethyl acetate ≥99.5% p.s.) was purchased from Carl Roth. Liquid chromatography was performed using silica gel 60 (0.04–0.063 mm (230–400 mesh)) purchased from Carl Roth. HPLC was performed using a Bischoff Chromatography Hyperchrome HPLC column 150 mm× 4.6 mm packed with Prontosil Kromaplus 100-5-C18 5.0 µm (Leonberg, Germany) in a Jasco System composed of: a Jasco DG-2080-53 3-Line-Degasser, a Jasco LG-980-02 ternary gradient unit, a Jasco PU-980 intelligent HPLC pump, a Jasco CO-2060Plus Intelligent column thermostat, a Jasco AS-1555 intelligent sampler, a Jasco UV-2077 multi-wavelength UV-VIS detector, and a Jasco LC-Net II/ADC. Jasco ChromPass Chromatography Data System Version 1.8.6.1 was used for control and analysis (Groß-Umstadt, Germany). Solvent A (H_2_O) was obtained using a Satorius Stedim Biotech Arium^®^ Pro VF (Goettingen, Germany). Solvent B (methanol (ultra) Gradient HPLC Grade) was purchased from J.T. Baker. (Center Valley, PA, USA). LC-MS was performed using a Bischoff Chromatography Hyperchrome HPLC Column 150 mm × 4.6 mm packed with Prontosil Kromaplus 100-5-C18 5.0 µm in an Agilent 1200 System (Santa Clara, CA, USA). To solvent A 0.1% formic acid (≥98%, p.a.; Carl Roth (Karlsruhe, Germany)) was added. Shaking of the micro-scale reactions was performed using an Eppendorf Thermomixer comfort (Hamburg, Germany) to define 20 °C and a Hettich Benelux MKR 23 (Geldermalsen, The Netherlands) to define 0 °C. Calculation of log*P* values was performed using ChemDraw Professional 16.0.0.82 (PerkinElmer, Waltham, MA, USA).

### 3.2. Methods

#### 3.2.1. Distillation of DADS

DADS is commercially only available at 80% purity. For further purification we used distillation under reduced pressure. To enhance the efficacy of distillation a Vigreux column (600 mm) was used. The crude DADS was stirred and tempered in an oil bath. The pressure was reduced to approximately 50 mbar. At an oil bath temperature of 120 °C the DADS fraction evaporated. The boiling point under these conditions was 80.5 °C. A purity of 98% was determined by HPLC. 

#### 3.2.2. Synthesis of Allicin without Pre-Formed Performic Acid

Distilled diallyl disulfide (DADS; 2 g, 13.7 mmol) was mixed in 5 mL formic acid and stirred for 5 min at 0 °C. H_2_O_2_ (30%; 3 mL, 29.6 mmol) was added slowly to the mixture. The reaction was stopped after approximately 4 h by addition of 25 mL distilled water and the mixture was extracted three times with DCM. The solvent was removed under reduced pressure and the product was dissolved in the eluent, a mixture of *n*-hexane and ethyl-acetate (2:1).

Separation was performed via liquid chromatography using 150 mm silica gel 60 in a column with a diameter of 30 mm. Fractions were collected into tubes cooled in an ice bath and TLC was used to identify fractions containing solely allicin. Those fractions were combined, dried over amorphous anhydrous sulfate (e.g., MgSO_4_ or CaSO_4_) and filtered. The solvents were removed under reduced pressure at RT to yield a clear, oily substance that smells like garlic. Yield: 1.64 g, 10.1 mmol, 74%.

^1^H-NMR (500 MHz, CDCl_3_): δ3.70–3.75 (m, 4H); 5.14–5.42 (m, 4H); 5.68–5.88 (m, 2H); ^13^C-NMR: (125 MHz, CDCl_3_) δ35.08, 59.82, 119.11, 124.10, 125.78, 132.8.

#### 3.2.3. Synthesis of Allicin Using Pre-Formed Performic Acid

Distilled diallyl-disulfide (DADS; 0.5 g, 3.5 mmol) was mixed in 2.5 mL methanol and stirred for 5 min at 0 °C. Performic acid solution (2.0 mL) (as described in Section 3.2.6.) was added slowly to the mixture. The reaction was quenched after 15 min by addition of 25 mL distilled water and the mixture was extracted three times with DCM. The solvent was removed under reduced pressure and the product was dissolved in a mixture of *n*-hexane and ethyl-acetate (2:1).

Separation was performed via liquid chromatography using 150 mm silica gel 60 in a column with a diameter of 30 mm and *n*-hexane and ethyl acetate (2:1) as eluent. Fractions were collected into tubes cooled in an ice bath and TLC was used to identify fractions solely containing allicin. Those fractions were combined, dried over an anhydrous sulfate, and filtered. The solvents were removed under reduced pressure at RT to yield a clear, oily substance that smells like garlic. Yield: 0.52 g, 3.204 mmol, 92%.

^1^H-NMR (500 MHz, CDCl_3_): δ 3.70–3.75 (m, 4H); 5.14–5.42 (m, 4H); 5.68–5.88 (m, 2H); ^13^C-NMR (125 MHz, CDCl_3_): δ35.08, 59.82, 119.11, 124.10, 125.78, 132.8.

#### 3.2.4. Synthesis of Mixed Thiosulfinates

Diallyl disulfide (DADS; 1 g, 6.84 mmol) and dipropyl disulfide (DPDS; 1g, 6.65 mmol) were mixed in 5 mL formic acid and stirred for 5 min at 0 °C. H_2_O_2_ (30%; 3 mL, 29.6 mmol) was added slowly to the mixture. The reaction was quenched after approximately 4 h by addition of 25 mL distilled water and the mixture was extracted three times with DCM. The solvent was removed under reduced pressure and the crude products were analysed by HPLC and HPLC-MS.

#### 3.2.5. Micro-Scale Reaction

DADS (10 mg, 68.4 µmol) was mixed in 25 µL of either formic or acetic acid in a 2.0 mL reaction tube on ice. The formic acid-containing tubes were placed in a cooling shaker at 0 °C, the acetic acid-containing tubes were placed in a shaker at 20 °C. Then H_2_O_2_ solution (30%, 15 µL, 148 µmol) was added to the mixture and the reaction was initiated by shaking at 1200 rpm. For sample collection, single tubes were removed and the reaction was quenched by diluting the mixture to 2 mL with methanol. The samples were stored at −20 °C prior to HPLC analysis.

#### 3.2.6. Performic acid Pre-Formation

If not explained differently, H_2_O_2_ and formic acid were mixed (in a ratio of 3:5) and incubated at RT for 90 min. In micro-scale reactions, for instance, 40 µL of that mixture was used instead of adding 25 µL formic acid and 15 µL H_2_O_2_.

#### 3.2.7. High-Performance Liquid Chromatography (HPLC) Analysis

Reaction mixtures were analyzed by loading each 20 µL sample onto the HPLC. Separation was performed using H_2_O as mobile phase A and methanol as mobile phase B with the following gradient: 56% A (pre-run); 53% A (10 min); 7% A (15 min); 7%A (30 min); 56% A (31 min); 56% A (35 min) at a flow rate of 1 mL/min and a column thermostat temperature of 25 °C. Under these conditions retention times were 4.8 min for allicin and 18.2 min for DADS. Byproducts appearing at 14.9 min and 17.7 min due to their calculated log*P* values are assumed to be forms of ajoene and vinyldithiine, but were not investigated further at this stage. To quantify allicin and DADS, external standards were used.

#### 3.2.8. Liquid Chromatography-Mass Spectrometry (LC-MS)

The LC-MS protocol used the same gradient and column as the HPLC protocol, except for the use of 0.1% formic acid, which was used instead of pure water. The following source conditions were employed: heater—350 °C; sheath gas flow rate machine settings (without units)—30; auxiliary gas flow rate—5; sweep gas flow rate—0; ion spray voltage—400 kV; capillary temperature—250 °C; capillary voltage—82,5 V; tube lens—120 V in a ThermoFischer LTQ XL (Waltham, MA, USA).

#### 3.2.9. Thin Layer Chromatography (TLC)

Approximately 2 µL of the reaction mixture was loaded on a silica plate. After drying, the substances were separated using *n*-hexane/ethyl-acetate mixture (in a ratio of 2:1) as mobile phase. Under these conditions spots were visible under UV light (254 nm). Allicin’s *R*_f_ value was 0.70 and DADS’s *R*_f_ value was 0.95.

## 4. Conclusions

Our data provide evidence that the reaction mechanism underlying the conversion of DADS to allicin in the presence of formic acid and H_2_O_2_ is similar to that already proposed by Nikolic, but without the need for hydroxyl radicals. The unpaired electrons in such radicals might delocalize and would surely result in a number of additional side products for which we see no evidence. As the four possible products from the mixture of disulfides (DPDS and DADS) were formed in approximately equal amounts, we surmise that direct oxidation of the disulfides without chain cleavage is probably not quantitatively significant and, thus, we suggest an oxidative cleavage mechanism for the reaction as shown in Scheme 3.

We also show that the optimized method we describe here to synthesize allicin is an improvement on the previously-published procedures based on the one of Lawson [10]. Not only does the utilization of formic acid as a catalyst lead to a purer product, since the formation of by-products is decreased, the reaction also occurs faster and is easier to perform under standard conditions. Formic acid offers another advantage during the purification of the product allicin because it is more volatile than acetic acid and therefore easily removed under reduced pressure. Other peroxy-acids such as magnesium monoperoxyphthalate, or chloroperbenzoic acid have also been used [11,12,13]. In light of economical reasoning, however, the price of formic acid compared to aromatic peroxy-acids is just another argument, which points to formic acid as the catalyst of choice for the synthesis of allicin. Additionally, formic acid, as a naturally-occurring organic molecule produced, for example, by red ants and stinging nettles, is more eco-friendly than most alternatives (with the possible exception of acetic acid) and certainly ‘greener’ than the aromatic alternatives. The use of silica gel chromatography offers the advantage whereby a separation of the product and byproducts can be achieved without further diluting the allicin excessively. Therefore, it is possible to continue the reaction until a maximal turnover is reached, purify the crude product, and obtain pure allicin rather easily. A suggested optimized protocol for the synthesis of allicin, taking into account the various individual improvements we describe here are, therefore, as follows:

1. Use redistilled DADS and add methanol to combine the aqueous and organic phases (see Section 3.2.3.) or keep the final reaction volume small to promote efficient mixing and achieve a high conversion rate.
2. Use formic acid as the acid catalyst and pre-form performic acid as described in Section 3.2.6.
3. Cool the reagents and carry out the reaction on ice.
4. Slowly add performic acid solution.
5. Continually stir the reactants as efficiently as possible and carry out the reaction at 0 °C for just 15 min.
6. Quench the reaction with water.
